# Historical samples reveal a combined role of agriculture and public‐health applications in vector resistance to insecticides

**DOI:** 10.1002/ps.6775

**Published:** 2022-01-19

**Authors:** Daniele Porretta, Valentina Mastrantonio, Valentina Lucchesi, Romeo Bellini, John Vontas, Sandra Urbanelli

**Affiliations:** ^1^ Department of Environmental Biology Sapienza University of Rome Rome Italy; ^2^ Medical and Veterinary Entomology Department Centro Agricoltura Ambiente ‘G. Nicoli’ Bologna Italy; ^3^ Department of Crop Science, Pesticide Science Lab Agricultural University of Athens Athens Greece; ^4^ Institute of Molecular Biology and Biotechnology Foundation for Research and Technology Hellas Heraklion, Crete Greece

**Keywords:** entomological collections, insecticide resistance, mosquitoes, vector‐borne diseases, integrated resistance management, diflubenzuron

## Abstract

**BACKGROUND:**

Insecticide resistance is the major threat to vector control and for the prevention of vector‐borne diseases. Because almost all insecticides used against insect vectors are or have been used in agriculture, a connection between agricultural insecticide use and resistance in insect vectors has been hypothesized. However, it is challenging to find a causal link between past agricultural use of insecticides and current resistance in vector populations without historical data series. Here we investigated the relative contribution across time of agricultural and public‐health insecticide applications in selecting for diflubenzuron (DFB) resistance in *Culex pipiens* populations. Using DNA sequencing, we looked for DFB resistant mutations in current and historical mosquito samples, dating back to the 1980s–1990s, when DFB was used in agriculture but not yet in mosquito control.

**RESULTS:**

In the samples collected before the introduction of DFB in vector control, we found the resistant mutation I1043M in rural regions but not any of the neighboring urban and natural areas, indicating that the selection pressure was derived by agriculture. However, after the introduction of DFB for vector control, the resistant mutations were found across all study areas showing that the initial selection from agriculture was further boosted by the selection pressure imposed by the mosquito control applications in the 2000s.

**CONCLUSIONS:**

Our findings support a combined role of agricultural and public‐health use of insecticides in vector resistance across time and call for specific actions in integrated resistance management, including increased communication between agriculture and health practitioners. © 2022 The Authors. *Pest Management Science* published by John Wiley & Sons Ltd on behalf of Society of Chemical Industry.

## Graphical abstract

Agriculture applications of diflubenzuron selected for resistance alleles in the vector *Culex pipiens* before the public‐health applications. Resistance was further boosted by the selection pressure imposed by the mosquito control applications in 2000s. These findings support the combined role of agricultural and public‐health use of insecticides in vector resistance across time.

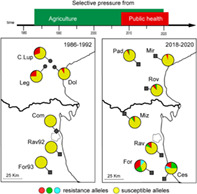

## 1 INTRODUCTION

Insect vectors are major threats to public health worldwide. Vector‐borne diseases transmitted by bloodsucking insects account for over 17% of all infectious diseases, and > 90% of this fraction is due to mosquito‐transmitted agents.^1^, ^2^, ^3^

Insecticides remain the most used tool in vector control, although the evolution of resistance poses a global challenge to their efficacy. In the last decades, resistance to insecticides has been documented in most insect species of public health importance.^4^, ^5^, ^6^, ^7^, ^8^, ^9^

Because many insecticide products are employed both in agriculture and public health, a connection between agricultural insecticide use and resistance in vector species has far been hypothesized.^10^, ^11^, ^12^, ^13^ Agricultural treatments can indeed put considerable selection pressure on non‐target vector populations present in the same fields. Several studies showed higher vector resistance in areas with agricultural insecticide use than in non‐agricultural areas. Likewise, reduction of vector densities following application of agricultural insecticides was documented.^12^, ^13^ While a correlation between current agricultural insecticide use and resistance in vector species can be easily detected by spatial correlation, finding a causal link between past agricultural use of insecticides and current resistance in vector populations is more difficult because data about the vector populations' resistance to an insecticide before its use in public health are usually not available.

The analysis of vector individuals preserved in historical collections that pre‐date the public‐health use of insecticides offers a unique opportunity to empirically evaluate the correlation across time between agriculture use of insecticides and resistance in vectors. Historical individuals keep genetic information from when they were sampled, therefore they can provide snapshots of genetic characteristics of populations over time.^14^, ^15^, ^16^

**Figure 1:**
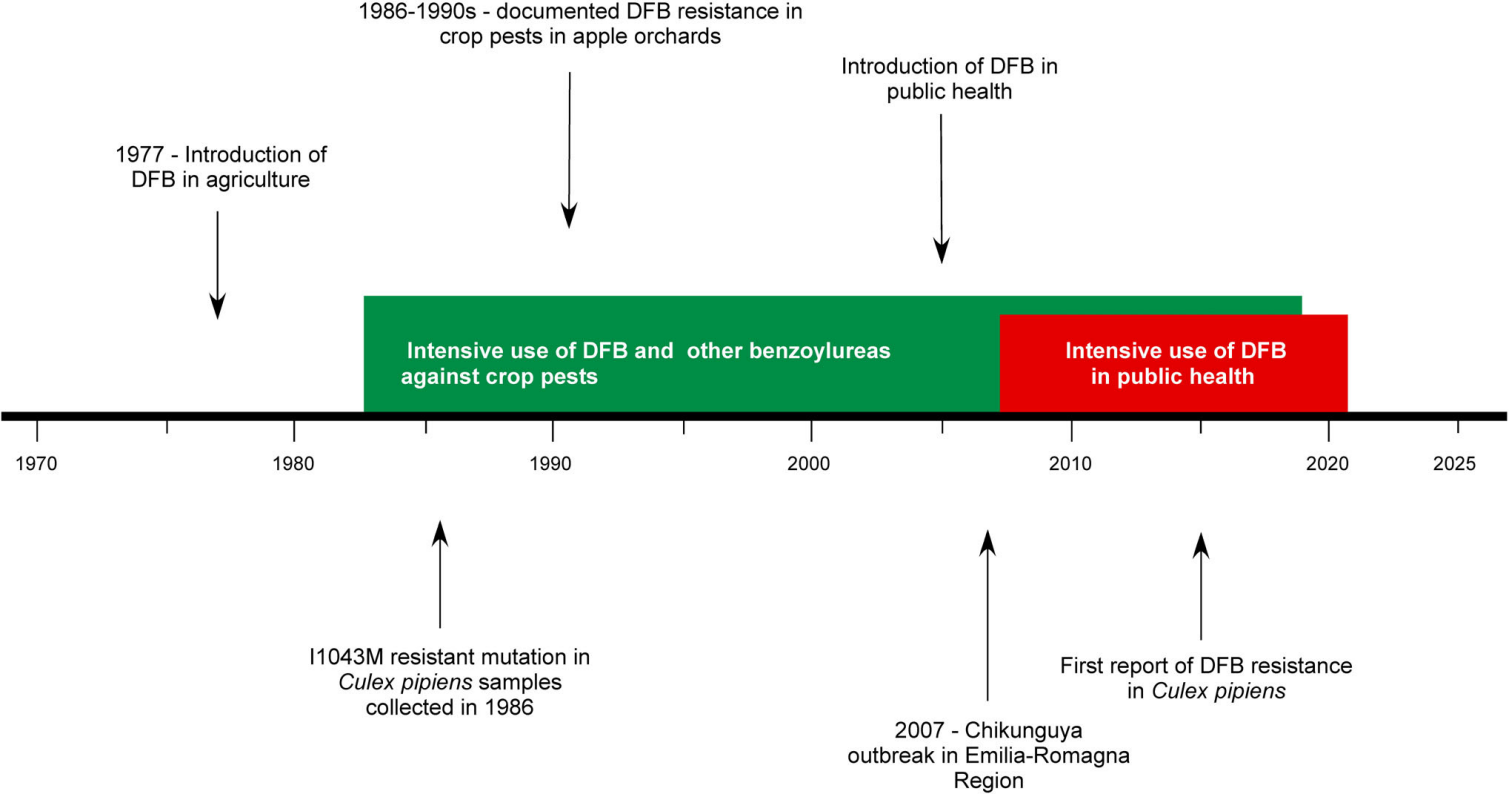
Diflubenzuron (DFB) use against orchard pests and mosquitoes in Italy.

In this article, we tested the utility of this approach, putting it in the case of the diflubenzuron (DFB) resistance in the mosquito *Culex pipiens* in northern Italy. DFB is a chitin‐synthesis inhibitor (CSI), interacting with the chitin‐synthase 1, the enzyme responsible for chitin synthesis in the cuticle.^17^, ^18^ In *Cx. pipiens*, DFB resistance was detected for the first time in 2015 in north‐eastern Italy.^18^ Resistance is associated with three point‐mutations in the codon 1043 of the chitin‐synthase 1 gene (*chs‐*1).^19^ The susceptible individuals carry the ‘ATC’ nucleotide sequence corresponding to the isoleucine amino acid (I1043). In contrast, the resistant individuals carry the mutations ‘CTC’, ‘ATG’ or ‘TTC’, corresponding to the leucine (I1043L), methionine (I1043M) or phenylalanine amino acids (I1043F), respectively.^18^, ^19^, ^20^, ^21^ DFB based products have been used intensively in north‐eastern Italy after the outbreak of Chikungunya virus in the Emilia‐Romagna region in 2007.^22^ However, before DFB was introduced in vector control, it was largely employed in agriculture from the 1980s to the early 2000s against orchard pests, such as the leaf miners and leafroller moths^23^, ^24^, ^25^ (Fig. 1). A possible association between DFB use in agriculture and insecticide resistance in *Cx. pipiens* populations has been therefore hypothesized.^26^

In this article, we aimed to investigate the relative role of selective pressure from agricultural and public‐health use of DFB in selecting for resistance in *Cx. pipiens* populations. At this aim, we analyzed both current and historical mosquito samples, dating back to the 1980s–1990s, when the DFB was used in agriculture, but not against mosquitoes. The detection of the resistant mutations in historical individuals sampled in rural areas should be regarded as direct evidence that agricultural treatments contributed to selection for resistant mutations before the DFB use in public health.^9^

## 2 MATERIALS AND METHODS

### 2.1 Study area and samples

*Culex pipiens* samples used in this study were collected in north‐eastern Italy in rural, natural and urban habitats. The study area is the eastern part of the Padana Plain, one of the most important agricultural areas in Italy. The landscape consists of several small urban areas surrounded by rural areas within a network of rivers and drainage canals. Agriculture, including fruit production, represents an important agricultural activity encompassing approximately a total of 50 962 ha (mainly apple, pear, peach, apricot, cherry).^27^ The area includes the Po River Delta, facing the Adriatic Sea, one of the largest wetlands along the Mediterranean coasts, characterized by rice paddies, lagoons and wooded wetland patches. In these habitats, *Cx. pipiens* is common and abundant, developing in temporary and permanent water sources, ponds, and in drainage and natural ditches.^28^, ^29^

**Figure 2:**
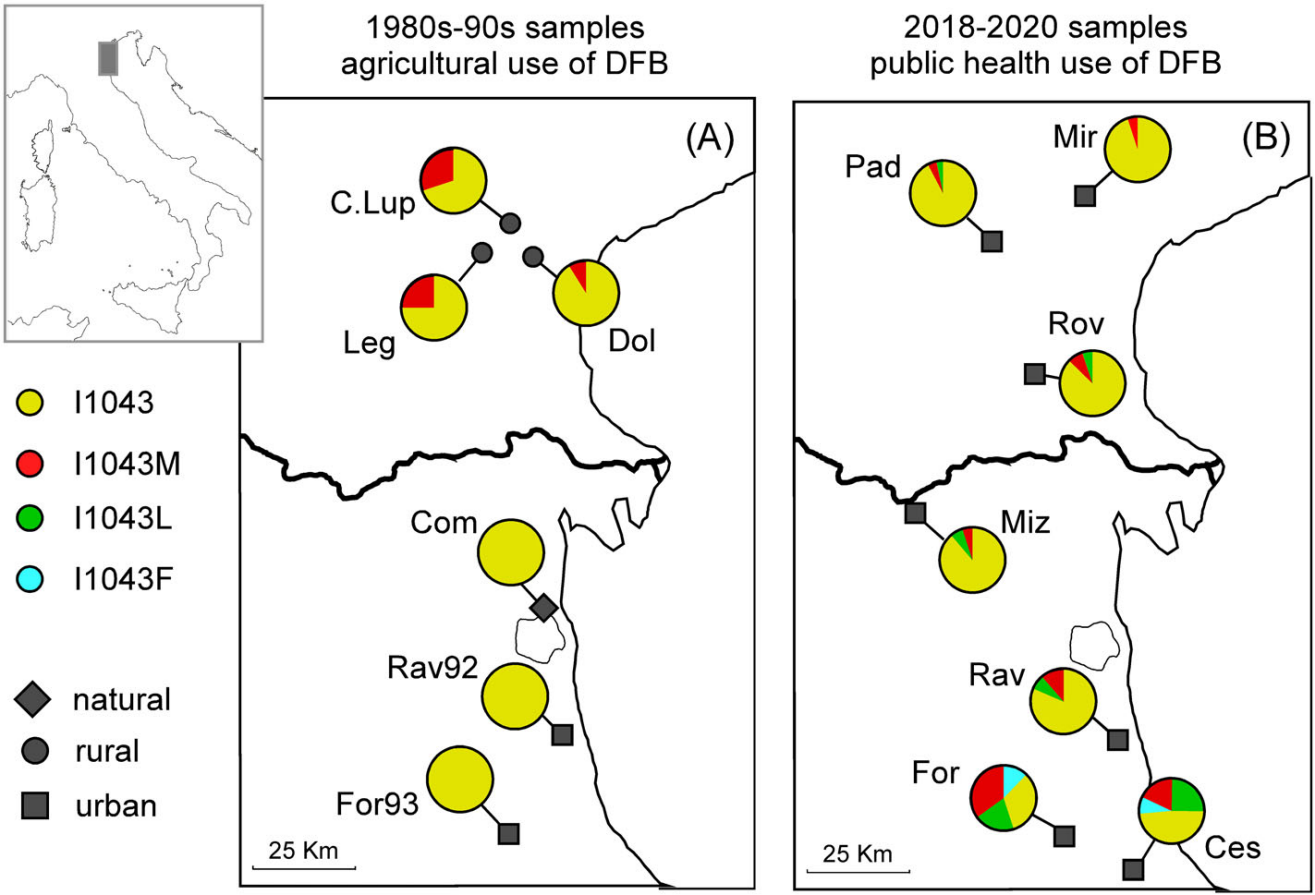
Distribution of diflubenzuron (DFB) resistance mutations in *Culex pipiens* populations. (A) Resistance frequency in samples collected in 1980s–1990s; (B) resistance frequency in samples collected in 2018–2020. The localities are encoded as shown in Table 1. For each locality, the frequency of the resistant and susceptible alleles, and the habitat characteristics of the sampling site are shown.

Both current and historical mosquito samples were analyzed. The current samples were collected in 2018–2020 in urban areas in the municipalities of Padova, Rovigo, Venezia, Ravenna, Ferrara and Forlì‐Cesena (Table 1 and Fig. 2). Mosquitoes were collected as larvae, identified morphologically to species^30^ and stored in ethanol 90% until genetic analyses.

**Table 1:** Collection sites of the *Culex pipiens* samples used in this study

Code	Locality	Municipality	Year	Habitat	No	1043 Allele frequencies			
						(I)	(L)	(M)	(F)
C.Lup	Campagna Lupia	Padova	1986	Rural	33	0.91	—	0.09	—
Leg	Legnaro	Padova	1986	Rural	36	0.81	—	0.19	—
Dol	Dolo	Padova	1987	Rural	35	0.74	—	0.26	—
Com	Comacchio	Ferrara	1987	Natural	42	1.00	—	—	—
Rav92	Ravenna	Ravenna	1992	Urban	37	1.00	—	—	—
For93	Forlì	Forlì‐Cesena	1993	Urban	24	1.00	—	—	—
Mir	Mirano	Venezia	2018	Urban	33	0.90	0.03	0.07	—
Pad	Padova	Padova	2018	Urban	48	0.98	0.01	—	0.01
Rov	Rovigo	Rovigo	2018	Urban	36	0.93	0.03	0.04	—
Miz	Mizzana	Ferrara	2020	Urban	37	0.93	0.06	0.01	—
Rav	Ravenna	Ravenna	2018	Urban	39	0.80	0.05	0.15	—
For	Forlì	Forlì‐Cesena	2018	Urban	33	0.38	0.20	0.32	0.10
Ces	Cesena	Forlì‐Cesena	2020	Urban	35	0.54	0.24	0.19	0.03

The frequency of the susceptible (I1043) and resistant alleles (I1043M, I1043L and I1043F) are shown for each locality.

The historical samples of *Cx. pipiens* used in this study were preserved at the Evolutionary Ecology Laboratory of the Sapienza University. They include mosquitoes from Legnaro, Dolo, and Campagna Lupia (Padova municipality) that were collected in 1986–1987 in apple orchards; samples from Comacchio, collected in 1987 in water ponds in wooded wetland south to the Po Delta; samples from Forlì and Ravenna, collected in urban areas in 1993 and 1992, respectively. After collection, mosquitoes were identified morphologically and stored at −80 °C.

### 2.2 DNA extraction

Genomic DNA was extracted from individual mosquitoes using the hexadecyl‐trimethyl‐ammonium bromide (CTAB) based method.^31^ Initially, mosquitoes were crushed with a pestle using liquid nitrogen. The powder was then placed into in 1.5 mL microcentrifuge tubes containing 200 μL of extraction buffer previously heated at 65 °C (5% CTAB, 1.4 mol/L NaCl, 0.2% 2‐β‐ mercaptoethanol, 20 mm EDTA, 100 mm Tris–HCl pH 8.0), and the tubes were incubated at 65 °C for 1 h. After cell lysis, proteins were removed with one volume of chloroform/isoamyl alcohol (24:1): the solutions were mixed by inversion for 5 min; the tubes were centrifuged at 13 000 rpm for 10 min and the upper aqueous phase was transferred into clean tubes. Next, DNA precipitation was carried out by adding one volume of isopropanol, mixing gently by inversion, and placing the tubes into a −20 °C freezer for 1 h. Finally, the pellet was washed with cold 70% ethanol. The DNA was re‐suspended in 30 μL of double distilled water and preserved at −20 °C. The concentration and quality of the extracted DNA were measured for each sample using NanoDrop ND‐1000 (Thermo Scientific, DE, Wilmington, USA).

To avoid cross‐contamination and contamination with exogenous DNA, genomic DNA extraction of current and historical samples was carried out in different laboratory rooms, and using different equipment exclusively maintained for working within such rooms; negative extraction was included to detect potential contamination in reagents and cross‐contamination between samples. Furthermore, extensive decontamination by ultraviolet (UV) radiation of the laboratory, working surfaces, and equipment were performed.

### 2.3 Polymerase chain reaction (PCR) amplification and sequencing

A fragment of the *chs‐*1 gene encompassing the 1043 codon was amplified by polymerase chain reaction (PCR). We first used the primer pairs described in Fotakis *et al*.^19^ to amplify a 700‐bp long fragment. However, the historical samples failed to be amplified, therefore, we used the I1043M‐F (5′‐GCCTGTCTCCATCCGCAAG‐3′) and I1043M‐R (5′‐CCCAGGAGACGACGTTCAG‐3′) primers that allow amplifying a fragment of about 130‐bp long.^18^ PCR amplifications were carried out in a 25 μL volume containing 5 ng of DNA, 0.4 μmol/L of the forward and reverse primers, 10 μL of 5× PCRBIO Reaction buffer (Resnova, Genzano, Italy), 0.5 μL of PCRBIO High Fidelity Polymerase (2u/μL) and water. As described earlier for DNA extraction, PCR amplification of current and historical samples were carried out in different laboratory rooms, using different equipment to avoid contaminations, and including negative controls to detect potential contamination in reagents.

PCR cycles consisted of 95 °C for 5 min, 30 cycles of 94 °C for 30 s, 60 °C for 30 s, 72 °C for 1 min and a final extension of 72 °C for 10 min. PCR products were run on 1.5% agarose, 0.5× TAE gel, and visualized by staining with Gelred (Sigma‐Aldrich, Milan, Italy) to check for quality. Finally, PCR products were double‐strand sequenced by standard Sanger sequencing by Microsynth Inc. (https://www.microsynth.ch.html). The sequences obtained were visualized with the software Chromas 2.6.5 (Technelysium, Helensvale, QLD, Australia) to check for sequence quality and resistant/susceptible genotypes. Samples with poor quality sequencing were excluded from the genotype/allele frequency analyses. All historical individuals showing resistant alleles (see Section 3) were re‐amplified and sequenced as described earlier, to further check for genotyping reliability.

## 3 RESULTS

A 130‐bp long fragment of the *chs‐*1 gene including the 1043 position was successfully amplified from current and historical *Cx. pipiens* mosquitoes and examined by sequencing for the presence of resistant mutations. The sequencing results showed the high reliability of this approach in discriminating susceptible and resistant genotypes. In the historical mosquito samples dating back to 1980s–1990s when the DFB were used in agriculture, but not against mosquitoes, the I1043M resistant mutation was detected in rural samples at a frequency ranging from 0.09 to 0.26. In the historical samples collected in natural (Comacchio) and urban areas (Ravenna and Forlì), no resistant alleles were detected (Table 1 and Fig. 2(A)).

In the samples collected in 2018–2020, the three I1043L, I1043F and I1043M resistant mutations were detected. The resistant allele I1043L was detected in all 2018–2020 *Cx. pipiens* populations, except the Mirano population, ranging from 0.01 to 0.24 frequencies (Padova and Cesena populations, respectively). The I1043F mutation was detected in Padova (0.01), Forlì (0.10) and Cesena (0.03) populations. The I1043M was detected in all populations except Padova, with a frequency ranging from 0.01 to 0.32 (Mizzana and Forlì population, respectively; Table 1 and Fig. 2(B)).

## 4 DISCUSSION

### 4.1 Combined selection from agricultural and public‐health applications

DFB was first commercialized in Italy in 1977 (N. Reg. 002454, Italian Ministry of Health). Since 1983 it was increasingly used in agriculture against orchard pests.^23^, ^32^ In 1986–1988 resistance to DFB was first found in crop pests in northern Italy orchards.^33^ During the 1990s resistance was increasing across Europe, and DFB use in orchards was reduced by the end of 1990s and finally replaced by other active ingredients.^24^, ^32^, ^34^, ^35^, ^36^ Notably, other benzoylureas targeting the chitin synthase 1,^37^ such as flufenoxuron, were still in use until 2011 against crop pests,^38^ while lufenuron could be used between 2011 and 2019 in Italy^39^ (Fig. 1).

In the early 2000s DFB was introduced in insect vector control. Its use in northern Italy greatly increased after the outbreak of Chikungunya virus in the Emilia‐Romagna region in 2007.^22^, ^40^ In the mosquito *Cx. pipiens*, DFB resistance was detected for the first time in 2015 in the Ravenna province.^18^ Further screening showed that resistant mutations are widespread along the eastern coasts of the Po Plain.^19^, ^21^, ^26^

Here, by analyzing historical collection individuals, we found the I1043M resistant allele in individuals sampled in 1986–1987 in three localities of northern Italy, which shows that this resistant allele was within *Cx. pipiens* populations before the DFB use in public health.

The I1043M allele was detected in historical samples only in the orchard samples, showing an association between the occurrence of resistance and agricultural areas where DFB was used (Table 1 and Fig. 2(A)). To date, the spatial association between insecticide applications in cultivated areas and vector resistance is regarded as the strongest evidence that the resistance is linked to insecticide agricultural use.^10^, ^11^, ^12^, ^13^ For example, in Côte d'Ivoire the populations of the malaria vector *Anopheles coluzzii* from agricultural areas where neonicotinoids insecticides are used exclusively for crop protection, are resistant to acetamiprid, while populations from non‐agricultural area are susceptible.^12^, ^13^, ^41^ In the *Cx. pipiens*, therefore, the usage of DFB and other benzoyloureas against crop pests could have exerted selection pressure on populations, and likely contributed to the maintenance of the I1043M resistant allele.

The initial selection from agriculture was further boosted by the selection pressure imposed by the mosquito control applications in 2000s. When DFB was introduced in public health, *Cx. pipiens* populations were pre‐adapted against this active ingredient, and resistant mutations further spread driven by the use of DFB against mosquitoes, resulting in the high mutation frequencies observed today.^19^, ^26^ Notably, the I1043L and I1043F mutations have not been found by our analyses of historical samples. They could have been at low frequency in the 1980s or could have originated *de novo* following the introduction of DFB in mosquito control.^9^, ^42^, ^43^

### 4.2 Applications to insecticide resistant management

Insecticide resistance is a long‐standing problem affecting the efficacy and utility of vector control compounds.^4^, ^5^, ^8^ Therefore, insecticide resistance management (IRM) is crucial. Using insecticide mixtures or rotating active ingredients are currently major approaches to prevent the emergence of resistance in susceptible populations, slow its evolution, or reverse it to a level compatible with efficient use of insecticides.^44^, ^45^ However, the panel of insecticides available for public health is very limited. The development of new molecules is an increasingly complex, long, and costly process that cannot be justified by vector control alone, representing < 1% of the total pesticide market. Therefore, almost all insecticides used for public health have been developed for agriculture and are (or have been) used for this purpose.^44^

By providing a link across time between agricultural and public‐health selection, our work calls for specific actions in IRM. Initially, the development and execution of IRM should consider carrying out a careful assessment of the historical use of active compounds in both agriculture and public health before their introduction in vector control programs. Likewise, resistance surveillance of the vector populations occurring in rural areas is crucial to detect resistant alleles to the insecticides used against crop pests. Finally, increased communication and collaboration between the agriculture and health practitioners on pesticides and resistance management issues is expected to increase knowledge transfer and better understand the links between agricultural chemicals and resistance in vector populations.

## AUTHOR CONTRIBUTIONS

DP, JV and SU conceived the ideas and designed the methodology; SU, RB collected historical samples; VM and VL collected present samples; VM and VL carried out DNA analyses; DP and VM analyzed the data; DP, JV led the writing of the manuscript. All authors contributed critically to the drafts and gave final approval for publication.

## CONFLICT OF INTEREST

The authors have declared that no competing interests exist.

## Data Availability

All data are included in the manuscript.
